# Heritable hazards of smoking: Applying the “clean sheet” framework to further science and policy

**DOI:** 10.1002/em.22412

**Published:** 2020-10-26

**Authors:** Abigail P. Bline, Kerry L. Dearfield, David M. DeMarini, Francesco Marchetti, Carole L. Yauk, Jill Escher, Workshop Presenters

**Affiliations:** ^1^ Fielding School of Public Health University of California Los Angeles Los Angeles California USA; ^2^ Burke Virginia USA; ^3^ Chapel Hill North Carolina USA; ^4^ Environmental Health Science Research Bureau, Health Canada Ottawa Ontario Canada; ^5^ Department of Biology University of Ottawa Ottawa Ontario Canada; ^6^ Escher Fund for Autism San Jose California USA

**Keywords:** epigenomics, genetic toxicology, germ cells, heritable effects, risk assessment, tobacco smoke

## Abstract

All the cells in our bodies are derived from the germ cells of our parents, just as our own germ cells become the bodies of our children. The integrity of the genetic information inherited from these germ cells is of paramount importance in establishing the health of each generation and perpetuating our species into the future. There is a large and growing body of evidence strongly suggesting the existence of substances that may threaten this integrity by acting as human germ cell mutagens. However, there generally are no absolute regulatory requirements to test agents for germ cell effects. In addition, the current regulatory testing paradigms do not evaluate the impacts of epigenetically mediated intergenerational effects, and there is no regulatory framework to apply new and emerging tests in regulatory decision making. At the 50th annual meeting of the Environmental Mutagenesis and Genomics Society held in Washington, DC, in September 2019, a workshop took place that examined the heritable effects of hazardous exposures to germ cells, using tobacco smoke as the example hazard. This synopsis provides a summary of areas of concern regarding heritable hazards from tobacco smoke exposures identified at the workshop and the value of the Clean Sheet framework in organizing information to address knowledge and testing gaps.

## 1 INTRODUCTION

Genotoxicity testing over the past five decades has focused primarily on somatic cells and the potential carcinogenic risk if any genomic damage was found. However, this was not always the case. Concern that some drugs and chemicals being introduced into the environment might cause germ cell mutation was the primary motivating factor in the 1960s for the establishment of mutagenicity testing and formation of the Environmental Mutagen Society, now the Environmental Mutagenesis and Genomics Society (EMGS) (DeMarini 2020). However, this focus shifted by the mid‐1970s to a concern for somatic cell mutagenesis and cancer due to the difficulty and expense of germ cell mutation assays and the development of the *Salmonella* (Ames) mutagenicity assay (Claxton *et al*. 2010; DeMarini 2020). Although no human germ cell mutagen has yet been declared by any national or international agency, data have accumulated that suggest that several environmental exposures, especially tobacco smoke, are likely human germ cell mutagens (DeMarini 2012; Beal *et al*. 2017; Marchetti *et al*. 2020).

A report from a meeting of the International Workshop on Genotoxicity Testing (IWGT) detailed heritable outcomes that need to be considered when asking what risk management questions must be addressed and what testing strategies are needed for risk assessment (Yauk *et al*. 2015). However, most regulatory bodies across the world do not have an absolute requirement to test drugs and environmental chemicals prior to approval for their ability to cause heritable damage or to assess the ramifications that such damage might have on human health (Cimino 2006). Furthermore, regulatory attention on genotoxicity has been based on a standard, one‐size‐fits‐all testing approach that does not incorporate risk specific to germ cells very well, as described further below.

Recently, an approach referred to as the “Clean Sheet” was proposed to encourage the examination of a substance's potential adverse effects based on its projected mode of action and anticipated human exposure versus relying exclusively on a predetermined set of standard assays (Dearfield *et al*. 2017; Luijten *et al*. 2020). This strategy emphasizes flexible and innovative exposure‐based approaches that analyze endpoints most relevant to human health risks. Broader application of the Clean Sheet approach can be facilitated by using case studies to explore this novel regulatory framework in different decision‐making contexts.

On September 19, 2019, a workshop entitled *Heritable Hazards of Smoking*: *Applying the* “*Clean Sheet” Framework to Further Science and Policy* was held in advance of the 50th Annual EMGS Meeting in Washington, DC. There is mounting evidence suggesting tobacco smoke exposure imparts heritable effects on germ cells with negative health implications for the resulting offspring (Beal *et al*. 2017). Given the historical prevalence of tobacco smoking, it is also a substance with a legacy of exposure that is carried among us today and will continue to be passed along to the next generations. Also, the availability of alternatives to traditional tobacco products (e.g., e‐cigarettes), which are increasingly being used by young people, means that exposure to tobacco and related products will likely remain a public health concern over the long term.

The workshop assembled researchers to present evidence regarding the genetic and epigenetic toxicity of tobacco smoke and related substances to germ cells, as well as the health effects identified in progeny born of those cells. In addition to exploring the utility of the Clean Sheet framework, the focus of this workshop was to heighten the awareness and urgency of heritable hazards via germ cell exposures and to encourage regulatory bodies to consider equally the health impacts to germ cells and somatic cells from exposures to environmental chemicals and pharmaceuticals.

## 2 APPLYING THE CLEAN SHEET FRAMEWORK TO THE CASE OF GERM CELL TOBACCO SMOKE EXPOSURE

The purpose of the Clean Sheet framework is to provide a systematic but flexible approach to risk assessment aimed at producing the most relevant and informative data for decision making (Dearfield *et al*. 2017). It details a series of steps that begins with identifying what the problem is and what risk management questions are most relevant to address (planning and scoping). During this step, the exposed populations and anticipated exposure levels are projected to initiate an exposure analysis. Next, the effort assembles what data, evidence, and information are already extant regarding the problem (build the knowledge base). After the knowledge base is assembled, the flexibility of the approach is brought into play with development of hypotheses about how the specific genetic endpoint being analyzed will lead to the adverse outcome of interest (create a rational biological argument). This step provides the basis for determining what genetic testing is most relevant to perform for risk assessment purposes (select assays, perform them, and review the results steps). The resulting data are then used to quantify risk, that is, dose–response analysis and selection of a point of departure (PoD) on the dose–response curve for extrapolation to human exposure levels. Once risk is characterized by combining the relevant testing data and exposure analyses, risk managers can use this information to develop actions to prevent or mitigate the adverse outcome.

In following the Clean Sheet framework, the workshop coordinators first undertook problem formulation and agreed that the current risk assessment paradigms fail to fully address the heritable consequences of environmental exposures (Table 1). The planning and scoping involved establishing the specific endpoints of concern: germ cell and heritable mutations, intergenerational phenotypic changes, and epigenetic effects transmitted across generations. Following this, a decision was made to use tobacco smoke as the case study for the problem, given the widespread population‐level exposure and extensive data. An expert group was then assembled to present the evidence and information associated with this exposure.

**TABLE 1:** Application of the Clean Sheet framework at the *Heritable Hazards of Smoking* Workshop

“Clean Sheet” approach steps	Workshop information/evidence
Planning and scoping (including anticipated exposure)	Somatic adverse effects from smoking are well established (e.g., cancer), but germ cell effects and effects on heritability awareness is very low Reason for workshop—Examine the evidence that exposure to tobacco smoke is a heritable concern, that is, to focus on heritable effects of germ cell exposures Brought together a diverse group of stakeholders (researchers, regulators, public interest groups, bioethicists) to share information Goal is to provide quantitative data to model the potential risk levels of substances that induce genomic damage and contribute to human adverse health outcomes If such data sets are not available or amenable to quantitation, then identify what needs to be done (e.g., fill data gaps) and whether there are alternative actions risk managers can take in lieu of setting regulatory limits Describe what exposed populations are at risk (e.g., young adults, fetuses) and discuss the potentially vulnerable periods of exposure to focus concern(s) Major risk management question ‐ what could assist regulatory agencies to act on the risk posed to germ cells?
Build knowledge base	Review current regulatory processes for germ cell mutagenicity Detail the unique features of germ cell biology and heritable effects (to assist targeting of appropriate studies to assay potential adverse effects) Review available evidence from recent key studies supporting the potential for heritable genetic and epigenetic effects of tobacco products
Create rational biological argument	Examine which studies can help to characterize the mode of action(s) of potential germ cell mutagens and thus provide insights on how to prevent damaging exposures Identify the most relevant pieces of available evidence that demonstrate that there is indeed a heritable risk due to tobacco smoke exposure What studies provide dose–response data for quantitative analysis and if not available, identify data gaps to fill
Select assays and perform them	Many assays provide qualitative information, but none identified for use in quantitating heritable risk Recommend assays that can be performed to address data gaps and to obtain useful data for quantitative analysis Further studies identifying the specific cellular and molecular mechanisms by which heritable effects are manifested to bolster the evidence and aid in creating a rational biological argument for heritable risk
Review results	Evidence strongly suggests that exposure to tobacco smoke can result in genomic and epigenomic changes to germ cells that are inherited and produce negative health effects in the resulting offspring
Select appropriate point of departure (for quantitative analysis)	The workshop did not identify studies that could provide a point of departure for performing quantitative analysis Recommendations for dose–response data to assist in identifying an appropriate point of departure
Determine expected exposure	The workshop focus did not include an exposure assessment, so quantitation of exposure was not discussed
Estimate acceptable levels for endpoints of human relevance	As there were no quantitative data and an exposure assessment, estimated levels could not be derived
Risk characterization	Recognize that over a billion people smoke cigarettes worldwide; current human exposure to tobacco smoke is extensive There are several lines of evidence that support tobacco smoke as a genotoxicant in germ cells Evidence indicates a combination of germ cell DNA/chromosomal damage, DNA mutations, and epigenetic alterations contribute to the observed effects Though quantitation of risk not possible at this time, evidence heavily suggests regulatory agencies need to focus on reducing harm to germ cells and mitigating heritable risk, particularly to young people and other vulnerable populations Alternate actions discussed in lieu of quantitative levels for regulatory consideration Effective communication strategies to better inform the public about heritable germ cell effects on health and consistent messaging are needed

At the workshop, experts first reviewed current regulatory processes for germ cell mutagenicity, considered the unique features of germ cell biology and heritable effects, and reviewed evidence from recent key studies supporting the potential for heritable genetic and epigenetic effects of tobacco products. A directed discussion with audience participation focused on key questions developed during the problem formulation. This discussion involved evaluating the weight of evidence, identifying data gaps and uncertainties, and interpreting the implications for risk assessment. Finally, ethical considerations and advocacy needs were examined in the context of risk assessment and regulatory action. Ultimately, the Clean Sheet framework was applied to the heritable risk of tobacco smoking with the goal of providing regulatory agencies with information on which to base future actions.

## 3 STATE OF REGULATION OF GERM CELL MUTAGENS

Given the far‐reaching health implications of germ cell mutations and other genomic changes (Stenson *et al*. 2017), identifying chemical agents that may produce such effects should be of high importance to regulatory agencies. However, standard toxicity testing paradigms are generally poorly designed to fully capture perturbations to germ cells that could result in heritable effects (Marchetti *et al*. 2020). This is in large part due to the limited window of development to which germ cells are typically exposed during testing, in addition to the fact that many assays expose only male germ cells, thus ignoring female germ cell susceptibility altogether. For instance, the in vivo rodent dominant lethal test (OECD 2016a) exposes adult male parents to a toxicant and measures embryonic lethality of their offspring halfway through gestation. Although this test would identify chemicals that cause severe genomic abnormalities during adult spermatogenesis that result in fertilization failures or embryonic death (Marchetti and Wyrobek 2005), it would not identify toxicants that cause non‐lethal mutations or epimutations. Furthermore, because the males are exposed only as adults, the test would not identify effects that result specifically from exposure to primordial germ cells (PGCs) or other early stages of germ cell development.

The mammalian spermatogonial chromosomal aberration test (OECD 2016b) likewise examines effects only during the mitotic proliferation of spermatogonia in adult animals, and there is no equivalent standard test for female germ cells. Similarly, the transgenic rodent gene mutation assay (OECD 2013) can be applied to identify chemicals that are mutagenic in male germ cells (Marchetti *et al*. 2018), but it is not applicable to female germ cells because not enough of them can be collected to conduct the assay. The lack of practical methods to assess mutagenicity in female germ cells was acknowledged as a critical research gap in the IWGT report (Yauk *et al*. 2015).

Under the global harmonized system, a substance may be classified as a Category 1A germ cell mutagen only if there is positive evidence from human epidemiological studies (UN 2017). However, these data are inherently difficult to produce given the implausibility of obtaining human populations exposed to a single toxicant, the invasive procedures required to obtain female germ cells or developing embryonic germ cells, and the technical limitations in identifying small genomic changes out of the 3 billion base pairs of the human genome. Consequently, no substances to date have been designated as Category 1A germ cell mutagens. However, this result is more likely a reflection of regulatory testing shortcomings than of biological reality.

At present, there is no standardized test guideline adequately designed to identify heritable effects from germ cell exposures, particularly those mediated through epigenetic changes. This omission means that there is essentially no place in the risk assessment process to account for such effects. Therefore, it is necessary for risk assessment paradigms to shift in ways that are better able to protect the health of present and future generations. This is where the Clean Sheet approach is most applicable because the hazards specific to germ cell biology necessitate different testing design from the standard genetic toxicity testing battery.

## 4 UNIQUE GERM CELL BIOLOGY

**FIGURE 1:**
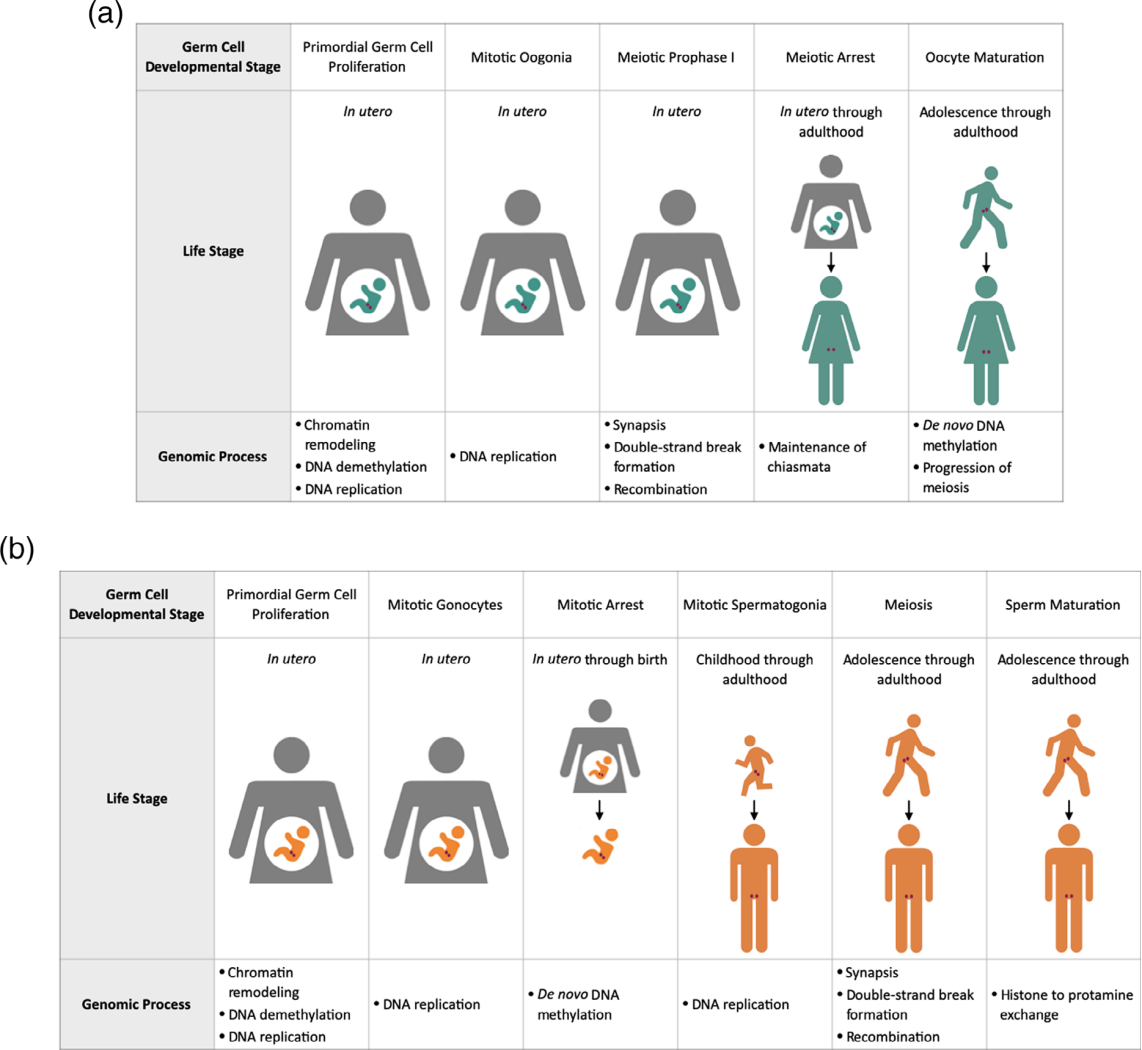
Overview of select developmental stages and genomic processes subject to environmental insults in (a) female and (b) male germ cells

To design relevant testing for germ cell damage, it is critical to understand the underlying biology of germ cell development (crucial for building the knowledge base). Using the knowledge of spermatogenesis and oogenesis can help researchers make better decisions regarding what testing assays to apply in determining when and how genomic damage to germ cells can occur (Figure 1). It also furthers the understanding of what chemical insults to germ cells mean in terms of adverse outcomes in exposed individuals. These are the types of data that are most useful for regulatory bodies to consider because they are most relevant to human heritable risk.

In humans, the earliest germ cells, PGCs, are first formed in the embryo approximately 2–3 weeks after fertilization (De Felici 2013). In both XX and XY embryos, the initial PGCs migrate to the genital ridge by the sixth week and continue mitotic proliferation through the 10th week post fertilization (Tang *et al*. 2016). After this time, germ cell development progresses in a sex‐specific manner.

In females, immature oogonia first enter meiosis at Embryonic Week 10 (Kurimoto and Saitou 2019). From this time until Embryonic Week 20, oogonia both proliferate via mitosis and progress through the diplotene stage of the first meiotic prophase before arresting in the dictyate stage (Sánchez and Smitz 2012; De Felici 2013). Now primary oocytes, these cells remain in the dictyate stage until puberty, after which time one primary oocyte per month resumes meiosis through the second meiotic prophase (Winship *et al*. 2018). The oocyte arrests again in this phase and completes meiosis only upon fertilization (Jones *et al*. 2013).

In males, the immature gonocytes continue mitotic proliferation until entering G0 mitotic arrest during the second trimester (Phillips *et al*. 2010; Kurimoto and Saitou 2019). Shortly after birth, gonocytes differentiate into primary A spermatogonia, which resume mitosis at age 5–7 years (Neto *et al*. 2016). Male germ cells first enter meiosis at puberty, when a subset of differentiated B spermatogonia give rise to spermatocytes that undergo meiosis and eventually produce mature spermatozoa (Neto *et al*. 2016). Throughout the rest of adulthood, male germ cells continue to undergo both mitosis to renew spermatogonial stem cells (SSCs) and proliferate spermatogonia, as well as meiosis to produce mature sperm.

As in somatic cells, mitotic germ cells are subject to DNA replication errors and chromosome missegregation, although they occur at relatively low rates under ambient conditions. Meiosis introduces a further level of risk for loss of genomic integrity due to the physiological induction of DNA double‐strand breaks (DSB) (Lindsey *et al*. 2013). DSBs allow for crossover events that facilitate genetic recombination and serve to tether homologous chromosomes (Winship *et al*. 2018). Genetic errors can be introduced during DSB repair or if repair fails to occur. Activation of cell‐cycle checkpoints can facilitate repair of DNA damage or trigger apoptosis if damage is too severe (Winship *et al*. 2018).

The ability of germ cells to cope with DNA damage is dependent upon both sex and developmental stage. For instance, fetal primary oocytes likely possess efficient DNA repair capacity but also appear to have a highly sensitive apoptotic response that correlates with the high rate of germ cell apoptosis that occurs prior to birth (Winship *et al*. 2018). During the protracted postnatal period of meiotic arrest, primary oocytes may be particularly sensitive to DNA damage but exhibit a decreasing ability to repair the damage with age (Myers and Hutt 2013). Oocytes also exhibit a relatively high incidence of aneuploidy (Jones *et al*. 2013) that substantially increases with increased maternal age at meiotic completion (Stuppia 2013).

Similar to fetal oocytes, postnatal gonocytes and spermatogonia appear to possess robust DNA repair capabilities (García‐Rodríguez *et al*. 2019). Apoptosis also serves as a quality‐control measure, with approximately 60% of differentiating B spermatogonia being eliminated in this way (Di Persio *et al*. 2017; García‐Rodríguez *et al*. 2019). However, mature spermatozoa have a limited capacity to perform DNA repair and are unable to complete apoptosis, which can result in the retention of spermatozoa with damaged and/or fragmented DNA (García‐Rodríguez *et al*. 2019). Additionally, the proportion of spermatozoa with DNA damage increases with age, which corresponds with a decrease in the rate of apoptosis (Singh *et al*. 2003). The DNA‐repair deficient phase of spermatogenesis represents a sensitive window for the accumulation of DNA damage that can persist and be transmitted by the sperm to the fertilized egg and result in genetic abnormalities impacting proper embryonic development (Marchetti and Wyrobek 2005).

In addition to DNA damage, the processes involved in germ cell specification and differentiation introduce periods of susceptibility to aberrant reprogramming of the epigenome. PGC specification is driven by expression of BLIMP1 and SOX17, which suppress somatic gene expression and promote germ cell gene expression, respectively (Tang *et al*. 2015). These transcription factors further lead to repression of the maintenance DNA methyltransferase DNMT1 and the de novo DNA methyltransferases DNMT3A and DNMT3B, as well as upregulation of TET1, which converts 5‐methylcytosine to 5‐hydroxymethylcytosine. As a result, global CpG methylation levels fall from approximately 80% at the time of PGC specification to approximately 5% in week 9 fetal PGCs (Tang *et al*. 2015). This demethylation facilitates parental imprint erasure as well as X chromosome reactivation.

Despite global demethylation, certain regions of the PGC genome are more resistant and retain their DNA methylation (Tang *et al*. 2015). In particular, evolutionarily young retrotransposons retain higher levels of methylation, which maintain their repression. In addition, approximately 6% of regions escaping DNA demethylation are depleted of repetitive elements and occur in genes with enhanced expression in the brain (Tang *et al*. 2015). These demethylation‐resistant genes, which are associated with diseases such as metabolic disorders, schizophrenia, and multiple sclerosis, may be important for epigenetic inheritance.

Although hypomethylation of gene promoter regions is generally associated with derepression of the gene, promoter methylation is decoupled from gene expression in PGCs, with only about 12% of hypomethylated genes exhibiting upregulation (Tang *et al*. 2015). These upregulated genes include those in the piRNA pathway as well as Krüppel‐associated box zinc finger genes that recruit repressive complexes to specific sequences, resulting in heterochromatin formation. Changes to posttranslational histone modifications during PGC development also serve to alter chromatin configuration (Tang *et al*. 2015). Therefore, chromatin reorganization adds an additional layer of transcriptional control during germ cell reprogramming.

Male fetal germ cells begin to regain DNA methylation in the second trimester after entering mitotic quiescence (Tang *et al*. 2015). De novo methylation appears to be largely completed within a few days after birth and, thus, prior to any meiotic activity (Wermann *et al*. 2010). Because de novo methylation occurs mostly in utero, male germ cell methylation marks may be highly influenced by the maternal environment. Conclusive studies on de novo DNA methylation of female germ cells have not been completed in humans (Yu *et al*. 2017). However, limited human data indicate that female germ cells remain hypomethylated until at least puberty (Wermann *et al*. 2010) and, similar to mice, de novo DNA methylation may coincide with the onset of oocyte growth (Sasaki and Matsui 2008).

In males, an additional epigenetic reprogramming event is the replacement of most histones with protamines during sperm maturation, which facilitates greater compaction of chromatin and results in a smaller nucleus. However, approximately 5–10% of histones are retained in mature sperm under physiological conditions (Lempradl 2020). Retained histones generally occur at the promoters of developmental genes as well as in areas of heterochromatin marked with H3K9 histone methylation (Lempradl 2020).

Germ cells undergo specific genomic and epigenomic processes during development that are distinct from somatic cells. Appropriately accounting for these differences is important for designing studies that are capable of determining windows of susceptibility when toxicant exposures are most likely to cause effects to germ cells that may impair fertility and/or be inherited by individuals descended from those cells.

## 5 EVIDENCE PRESENTED AT THE WORKSHOP

The biological knowledge described above set the stage for the interpretation of results from different studies, and the Clean Sheet provided a framework for considering them regardless of whether they were conducted following established test guidelines or not. The data from such studies can help to characterize the mode of action(s) of potential germ cell mutagens and, thus, provide insights on how to prevent damaging exposures (Yauk *et al*. 2013; Sasaki *et al*. 2020). It should be noted that the evidence summarized below should not be construed as a comprehensive review of the existing literature. Rather, presenters described empirical data from key studies in both animal models and humans (epidemiological studies) on the effects of tobacco smoking directly on germ cells or on the offspring of smokers. In pregnant females who smoke, both the somatic and germ cells of the F_1_ generation are exposed, with potential heritable effects manifesting in the F_2_ generation. In adult female smokers who cease smoking prior to conception, impacts on their own oocytes manifest in the F_1_ generation. In the case of paternal smoking, germline‐mediated effects are evident in the F_1_ generation. The presenters cautioned that these studies presume germ cell exposure through the smoker, not secondhand or sidestream smoke.

### 5.1 Effects of smoking on the germ cell genome

The available evidence on the genomic impact of tobacco smoking in human germ cells was reviewed recently by Beal *et al*. (2017); here, we briefly summarize some of the main findings presented in that comprehensive review. In humans, tobacco smoking was associated with (a) an increase by 1.2‐ to 5.0‐fold in the incidence of DNA breaks in sperm as measured by the TUNEL and comet assays; (b) an increase in the incidence of sperm aneuploidies for all chromosomes that have been investigated, with effect sizes ranging from 1.6 to 3.0; and (c) an increase by 1.2‐ to 7.6‐fold in the formation of sperm DNA adducts caused by a metabolite of benzo[*a*]pyrene (B[*a*]P), a known human carcinogen and main component of tobacco smoke. In mice, cigarette smoke exposure has been found to significantly increase the frequency of tandem‐repeat mutations in SSCs. This effect was found with both mainstream and sidestream smoke exposure. When exposed to B[*a*]P in utero, F_1_ mice were found to exhibit an increased mutation frequency in their sperm (Meier *et al*. 2017).

Less is known about direct effects of tobacco smoke on female germ cells. However, according to a review by Zenzes (2000), smoking may decrease the quality and quantity of oocytes. For example, smoking was associated with an 8–17% reduction in oocytes retrieved during in vitro fertilization, with higher smoking rates corresponding with a greater reduction. The effect of smoking upon oocyte number was further compounded by increasing age. Conversely, a dose‐dependent relationship was found between increased smoking and earlier age of menopause, which is caused in part by oocyte depletion. A dose‐dependent relationship has also been found between maternal smoking and incidence of spontaneous abortion, further suggesting smoking may cause genomic aberrations in female germ cells.

### 5.2 Effects of smoking on the germ cell epigenome

The effects of smoking on the germ cell epigenome have been evaluated by examining changes in DNA methylation levels across the genome. Based on one of the largest studies of DNA methylation patterns in the sperm of adult males by Jenkins *et al*. (2017), smoking did not appear to correlate with global changes in methylation levels; however, it did appear to be associated with an increase in the variability of methylation levels. Differential methylation appeared to be enriched at sites reported to escape protamine replacement during spermatogenesis, suggesting that DNA with retained histones may be more sensitive to environmental insults. This study also demonstrated that CpG sites that are resistant to demethylation during germ cell reprogramming appeared to be overrepresented in the differentially methylated sites.

In laboratory studies on mice, nicotine alone has also been found to induce DNA methylation changes in sperm. According to a study by McCarthy *et al*. (2018), the sperm of mice exposed to nicotine via drinking water exhibited a global increase in DNA methylation levels. However, DNA methylation levels specifically in the promoter region of the D2 dopamine receptor were decreased. This finding corresponded with a significant decrease in D2 receptor mRNA expression in the striatum of male F_1_ mice descended from nicotine‐exposed fathers. Although there were tissue‐ and sex‐specific differences in the effects observed in the F_1_ generation, these data suggest that nicotine may be capable of inducing epimutations in sperm that affect the phenotype of the resulting offspring.

### 5.3 Germ cell‐mediated effects of smoking on offspring genome

A few human studies provide evidence supporting an impact of paternal tobacco smoking on the offspring genome. One small human study by Linschooten *et al*. (2013) indicated that paternal smoking within 6 months of conception correlated with a significant increase in tandem repeat minisatellite mutations in their offspring. Furthermore, the effect appeared to be dose dependent, with 5.3, 19, and 33% of children from nonsmoker, irregular smoker, and daily smoker fathers, respectively, exhibiting mutations. An additional human study by Laubenthal *et al*. (2012) found that paternal preconception smoking was positively associated with single‐ and double‐strand DNA breaks in the cord blood of their offspring. These human findings are complemented by a recent mouse study by Beal *et al*. (2019) showing that paternal exposure to B[*a*]P significantly increased copy number duplications and de novo mutations in the offspring. Thus, there is growing evidence that paternal tobacco smoking and exposure to the components of tobacco smoke lead to an increase in the number of mutations that offspring inherit.

### 5.4 Germ cell‐mediated effects of smoking on offspring epigenome

The impact of tobacco smoking on the offspring epigenome is less established. However, recent studies provide support for epigenomic alterations. In a human study by Knudsen *et al*. (2019), whole blood samples were collected from adolescent and adult F_1_ offspring to examine the effects of paternal (F_0_) smoking on DNA methylation. This study identified differentially methylated regions associated with paternal smoking in genes related to innate immune system pathways as well as lipid metabolism and fatty acid biosynthesis. The study was unable to conclusively determine if the observed effects were due to altered methylation in the fathers' sperm because it is possible that secondhand smoke during gestation and/or childhood could have contributed to these changes. However, it was notable that persistent DNA methylation changes were discernible in adult F_1_ offspring up to 54 years of age.

Based upon a separate study by Joubert *et al*. (2016) of cord blood collected from 6,685 newborns, maternal smoking was associated with significant differences in DNA methylation of the aryl hydrocarbon receptor repressor gene. However, because this study included women who continued to smoke during pregnancy and, thus, during somatic development of the newborns, it cannot be determined how many of these methylation changes, if any, were attributable specifically to changes induced in the maternal germ cells.

### 5.5 Germ cell‐mediated effects of smoking on offspring phenotype

Evidence is accumulating that preconception tobacco smoking can result in a variety of phenotypic outcomes in the offspring. For instance, paternal cigarette smoking appears to impair the fertility of their male offspring. Specifically, Axelsson *et al*. (2018) found that paternal smoking near the time of conception correlated with a 41% decrease in sperm concentration and a 51% decrease in sperm count in the adult sons of smoking fathers. A more recent study by Haervig *et al*. (2020) with a larger cohort using data from the Danish National Birth Cohort produced similar findings.

Two types of childhood cancers appear to exhibit elevated risk from germ cell exposures to tobacco smoke. Pang *et al*. (2003) reported that leukemia risk was elevated in children of fathers who smoked prior to conception. They also found that hepatoblastoma risk was elevated with both paternal and maternal preconception smoking, and this risk was compounded further when both parents smoked prior to conception. These and other supporting data were considered sufficient by the International Agency for Research on Cancer to conclude that paternal tobacco smoking is a causative factor for an increased risk of childhood cancer (IARC 2012).

Asthma is another disease linked to germline exposure to tobacco smoke. One large cohort study by Accordini *et al*. (2018) utilized data collected from 2,233 mothers and 1,964 fathers to examine the associations between parental and grandparental smoking and asthma risk in offspring. In the case of offspring (F_2_) whose mothers (F_1_) were exposed to maternal (F_0_) smoking in utero, there was a nonsignificant relative risk ratio (RRR) of 1.3 for asthma. The RRR was lower for offspring (F_2_) of fathers (F_1_) exposed to maternal (F_0_) smoking in utero. However, a significant increase in asthma was found for offspring (F_2_) of fathers (F_1_) who started smoking prior to the age of 15 years, with an RRR of 1.43. This elevated risk was eliminated if the father began smoking after 15 years of age. A previous study by Svanes *et al*. (2017) had found similar results, with significant increases in risk for childhood asthma associated with grandmaternal smoking as well as with paternal smoking starting before the age of 15 years.

An earlier, smaller cohort study by Li *et al*. (2005) found a higher risk of asthma associated with grandmaternal smoking compared to the Accordini et al. study. In offspring (F_2_) whose grandmothers (F_0_) smoked while pregnant with their mothers (F_1_), there was an odds ratio (OR) of 2.1 for developing childhood asthma. No elevated risk of asthma was found for offspring (F_2_) whose mothers (F_1_) quit smoking prior to pregnancy.

Cohort studies have indicated that grandparental smoking also elevates the risk of autism, autism traits, and attention‐deficit/hyperactivity disorder (ADHD). A study by Golding *et al*. (2017) using data from the Avon Longitudinal Study of Parents and Children cohort showed significantly elevated odds for autism traits and diagnosed autism in grandoffspring (F_2_) of pregnant smokers (F_0_). A separate study by Yim (2019) using data from the Nurses' Health Study II found that grandmaternal (F_0_) smoking was associated with an adjusted OR of 1.18 (95% CI, 1.11, 1.25) for ADHD in the F_2_ generation.

Animal studies indicate an elevated risk for asthma from germ cell exposure to nicotine alone. In a multigenerational study by Rehan *et al*. (2012), the F_2_ offspring of F_1_ rats exposed to nicotine both in utero and for 21 days postnatally exhibited significant decreases in pulmonary function, indicative of an asthma‐like phenotype. Because the F_2_ generation was exposed to nicotine via the germ cells developing in the F_1_ generation, the phenotypic response in the F_2_ generation was mediated likely by germ cell changes. In a follow‐up study (Rehan *et al*. 2012), this asthma‐like phenotype was found to persist into the F_3_ generation, further supporting the possibility of germline inheritance.

Mouse studies indicate that both grandmaternal and paternal exposure to nicotine alone can also increase ADHD‐like phenotypes in offspring (Zhu *et al*. 2014; McCarthy *et al*. 2018). In the former case, Zhu *et al*. (2014) found that the F_1_ offspring of female (F_0_) mice exposed to nicotine for 3 weeks prior to mating and for the duration of pregnancy exhibited ADHD‐like behaviors in both sexes. However, only F_2_ mice descended from F_1_ females continued to exhibit these behaviors. The behaviors were also found in F_3_ mice descended from F_2_ females. Similarly, a study by McCarthy *et al*. (2018) from the same lab found that when adult male (F_0_) mice were exposed to nicotine, both male and female F_1_ mice exhibited ADHD‐like behaviors.

Overall, the evidence presented at the workshop strongly suggests that exposure to tobacco smoke can result in genomic and epigenomic changes to germ cells that are inherited and produce negative health effects in the resulting offspring. However, despite the breadth of information provided, the workshop attendees agreed that additional pieces of information are required to further the risk assessment of tobacco smoke exposure to germ cells.

## 6 DATA GAPS IDENTIFIED AT THE WORKSHOP

To perform a quantitation of effects for germ cells, a dose–response analysis of the relevant test results is needed. However, one of the greatest omissions in most studies examining effects of tobacco smoke on germ cells is demonstration of a clear dose–response relationship. Most epidemiological studies class people either as never or ever smokers with little differentiation by duration or intensity of smoking. In animal studies, only one dosing condition is often tested. Because toxicology is classically founded in the principle that “the dose makes the poison” (although nonmonotonic responses should not be precluded and in the context of germ cells, the developmental window of exposure is likely an important determinant of effect), evidence of a clear dose response would strengthen the evidence of heritable germ cell impacts of smoking. In addition, such an analysis would provide the basis for a quantitation of risk.

Similarly, further studies identifying the specific cellular and molecular mechanisms by which heritable effects are manifested would bolster the evidence and aid in creating a rational biological argument for heritable risk. However, a major complicating factor with evaluating risk from tobacco smoke is that it is a complex and variable mixture with over 4,000 individual compounds. Any number of these compounds could lead to various molecular initiating events that could synergize, antagonize, or otherwise interact with one another. Furthermore, products such as e‐cigarettes are increasingly being used as alternatives to tobacco cigarettes, particularly among young people (Jaspers 2019). These products present their own unique combinations of active and inactive ingredients that require specific attention in the risk assessment process.

Beyond mixture toxicity, more concerted study of mechanisms based upon germ cell biology could better inform which stages of germ cell development are most sensitive to tobacco smoke exposure. Studies specifically examining the link between genomic and/or epigenomic changes in parental germ cells to phenotypes observed in offspring and grandoffspring are lacking. Although many of the phenotypic outcomes reported in offspring are likely due to epigenomic changes in parental germ cells, this link has not been explicitly examined in a meaningful way. Information regarding epigenomic changes in germ cells that may be inherited in offspring are especially needed. The question of how many generations through which such epigenetic changes may persist also needs to be addressed systematically.

## 7 IMPLICATIONS FOR RISK ASSESSMENT

Risk assessment should be focused on addressing the most appropriate risk management questions relevant to human health outcomes (Dearfield *et al*. 2017). The subject workshop sought to address the question of the heritable risk posed to germ cells by exposure to tobacco products (see Table 1), which is of high importance for population health but has not been accounted for specifically in standard risk assessment processes. Given that 1.1 billion people smoke cigarettes worldwide (WHO 2019a), current human exposure to tobacco smoke is extensive. Furthermore, this number does not include individuals who may be exposed currently to secondhand/sidestream smoke or those who were exposed ancestrally, either from parental or grandparental smoking going back several decades. Therefore, the vast scale of human exposure, including germ cell exposure during various potentially susceptible windows of development, supports the need for further assessment.

As summarized in Table 1, the workshop made substantial progress in assessing the heritable risk of tobacco product exposure to germ cells using the Clean Sheet framework. As was shown during the workshop, there is a wealth of qualitative information supporting the biological argument that tobacco smoke can alter the genome and epigenome of both nascent and adult germ cells. However, the available data for tobacco smoke exposure are not sufficient for quantitating the risk of adverse health effects in the offspring of smokers, although certain components of tobacco smoke may provide some useful information. Based on the evidence presented and the data gaps identified above, the key information needed now is (a) human data directly linking parental tobacco smoking status, germ cell damage, and offspring phenotype in a dose‐responsive manner (e.g., number of cigarettes, years of smoking) and (b) mechanistic data to better define the adverse outcome pathway (AOP) by which such heritable effects occur.

Conducting a large, multigenerational cohort study designed specifically toward these ends would be one of the most valuable steps in advancing research on the heritable effects from exposure to tobacco smoke. Several large cohort studies already exist in multiple countries, and the available data could be readily leveraged for this purpose. Specifically, a human study investigating the F_2_ health effects from F_0_ grandmaternal smoking where the F_0_ smoking dose is known would improve our understanding of heritable effects established during PGC development (Escher and Robotti 2019). Multiple phenotypic endpoints in the F_2_ generation should be evaluated, including those related to cardiopulmonary, allergy/asthma, metabolic, and neurodevelopmental disorders and diseases. To assist in mechanistic understanding, genomic and epigenomic data from the F_0_, F_1_, and F_2_ generations should also be collected to the extent feasible. Multigenerational animal studies could assist in obtaining additional molecular data, although differences in gametogenesis and development must be thoughtfully accounted for in study design.

Because the available evidence indicates that sex and developmental differences in germ cells may also alter their susceptibility to genomic damage, it would be reasonable to conduct additional studies of paternal smoking with similar endpoints analyzed. In particular, F_0_ paternal smoking before the age of 15 appears to increase F_1_ asthma risk (Svanes *et al*. 2017). Thus, further studies to examine this time as a particularly sensitive window for male germ cell exposure to tobacco smoke are warranted.

Information from such studies could then be used to determine a PoD for germ cell genomic damage from tobacco smoke; see Johnson *et al*. (2014) for PoD determination. Combined with existing exposure data, the PoD data would allow for development of risk estimations for use in regulatory decision making (Dearfield *et al*. 2017) in those jurisdictions where it is appropriate to do so. We note that the Clean Sheet framework can be applied to any relevant human exposure (e.g., air pollution) to ensure that regulatory action adequately accounts for the risk specific to heritable germ cell effects. Until a formal accounting of the risk is completed and regulatory action is implemented, as appropriate, potentially heritable germ cell damage from relevant human exposures may continue to be inflicted today and experienced by multiple generations in the future.

## 8 ETHICAL CONSIDERATIONS AND ADVOCACY NEEDS

Ethical considerations, advocacy, and communication needs were major topics of discussion at the workshop after the scientific evidence for heritable risk from tobacco smoke was presented. Germline effects of tobacco exposure raise novel, intergenerational ethical issues (le Goff 2019). Not only may individuals whose germ cells are exposed to genotoxic agents suffer reproductive health consequences, but also individuals conceived from these germ cells may bear severe developmental and health consequences. The deferred nature of effects inherited from germ cells deeply challenges our framework for moral accountability because future generations consisting of people yet to be conceived do not have rights in the same way existing humans do. Although causation between present health outcomes and ancestral exposure is extremely difficult to establish, when causation can be demonstrated, it creates a responsibility in the present for effects that can be expected to occur in future generations.

The body of evidence presented at the workshop unequivocally shows that the risk that tobacco smoke poses to germ cells is substantial, even if not yet precisely quantified. From a bioethics standpoint, knowledge of a significant risk that contemporary actions impose onto future generations is sufficient to entail responsibility on the part of tobacco corporations, regulatory bodies, and individual smokers (le Goff 2019). Steps to assume this responsibility include (a) the study and incorporation of germ cell effects, in addition to individual or fetal health effects, into the assessment of tobacco smoke toxicity; (b) the regulatory limitation of availability and access of tobacco products; and (c) legal and social efforts to hold corporations accountable.

Beyond efforts to contribute to the improvement of regulation, researchers need to communicate their science in a clear and meaningful way to the general public and decision makers alike. Although most people are aware that tobacco smoke is bad for their own health, and some may perceive fetuses as additionally subject to harm, it is much less likely that people would consider impacts to their germ cells in particular and the potential consequences for future generations. Confining this knowledge to the scientific community without a concerted effort to share it more widely with the general public does a disservice to society and could be construed as morally irresponsible.

## 9 WORKSHOP CONSENSUS

The *Heritable Hazards of Smoking* workshop was convened on the premise that current risk assessment paradigms are unable to account for heritable effects from chemical exposures of germ cells and, therefore, cannot be relied upon to guide appropriate regulatory decisions. The Clean Sheet framework was employed as a tool to better articulate the problem and provide direction for future actions in the interest of improving public health.

During the workshop, a wide range of evidence was presented indicating that tobacco smoke can cause genomic and epigenomic alterations to germ cells, resulting in impaired fertility as well as negative health effects in subsequent generations. However, it was widely acknowledged that additional studies that demonstrate a dose response and provide more information on molecular mechanisms are necessary to aid in the development of an AOP and provide a more compelling argument for regulatory action. Therefore, future research should prioritize addressing these gaps through use of large cohort data, modern omics technologies, and targeted animal studies.

In addition to advancing the science, researchers should improve their communication of the science to the public. Raising awareness about concerns for heritable germ cell effects among those beyond the research community would help to both inform individual decision making and build a larger base of support for regulatory change.

Although the data are insufficient for quantitative risk purposes at the present time, based on the evidence presented, there are many other actions regulatory agencies can take to protect individuals from tobacco‐related harm and subsequent health risks; see Codex Alimentarius (2015) for information on risk management options from risk assessment outcomes. An important action already taken is the labeling of tobacco products that warn of health risks, although the messaging specific to heritable effects should be more prominent.

Guidance to particularly susceptible populations to avoid exposure would also be extremely useful. Young people as a susceptible population were discussed at length during the workshop. Based on the biology of oogenesis and spermatogenesis, there are a multitude of opportunities for deleterious genetic and epigenetic effects on the germ cells of exposed humans. Exposures to young people, while their germ cells are still developing, can be particularly deleterious. Effective measures to minimize the risk posed to their genetic material are needed from regulatory agencies.

Although tobacco smoke was the primary focus of the workshop, the workshop acknowledged that there are emerging concerns about germ cell harms from exposures to related substances such as e‐cigarettes, smokeless tobacco, and cannabis. Although the general health concerns surrounding cigarette smoking have been promoted widely and internalized by the public, these alternative products may be seen as relatively harmless, particularly among young people (Jaspers 2019). Thus, as the public health ramifications of cigarette smoking are abating with reduced usage (WHO 2019b), a new wave of current and future health impacts may be rising with increased usage of these alternative products. In response, regulatory agencies should take a more proactive and comprehensive approach to mitigating the potential risk to avoid a new health crisis.

Regardless of the substance, hazards to germ cells and the progeny to which they give rise must be accounted for explicitly in chemical risk assessment and regulatory decision making. Insults to the genomic integrity of germ cells introduce a significant risk to the welfare and health of people who inherit these genomic changes. If we continue to ignore these risks, we may be jeopardizing irreversibly human health and wellbeing for generations.

## CONFLICT OF INTEREST

The authors declare no conflict of interest.

## AUTHOR CONTRIBUTION

All authors contributed to the organization of workshop, drafted the manuscript, provided critical revisions, and approved the final manuscript.
